# Decreased Integrity, Content, and Increased Transcript Level of Mitochondrial DNA Are Associated with Keratoconus

**DOI:** 10.1371/journal.pone.0165580

**Published:** 2016-10-26

**Authors:** Xiao-Dan Hao, Zhao-Li Chen, Ming-Li Qu, Xiao-Wen Zhao, Su-Xia Li, Peng Chen

**Affiliations:** 1 State Key Laboratory Cultivation Base, Shandong Provincial Key Laboratory of Ophthalmology, Shandong Eye Institute, Shandong Academy of Medical Sciences, Qingdao, China; 2 Shandong Eye Hospital, Shandong Eye Institute, Shandong Academy of Medical Sciences, Jinan, China; Ben-Gurion University of the Negev, ISRAEL

## Abstract

Oxidative stress may play an important role in the pathogenesis of keratoconus (KC). Mitochondrial DNA (mtDNA) is involved in mitochondrial function, and the mtDNA content, integrity, and transcript level may affect the generation of reactive oxygen species (ROS) and be involved in the pathogenesis of KC. We designed a case-control study to research the relationship between KC and mtDNA integrity, content and transcription. One-hundred ninety-eight KC corneas and 106 normal corneas from Chinese patients were studied. Quantitative real-time PCR was used to measure the relative mtDNA content, transcript levels of mtDNA and related genes. Long-extension PCR was used to detect mtDNA damage. ROS, mitochondrial membrane potential and ATP were measured by respective assay kit, and Mito-Tracker Green was used to label the mitochondria. The relative mtDNA content of KC corneas was significantly lower than that of normal corneas (*P* = 9.19×10^−24^), possibly due to decreased expression of the mitochondrial transcription factor A (*TFAM*) gene (*P* = 3.26×10^−3^). In contrast, the transcript levels of mtDNA genes were significantly increased in KC corneas compared with normal corneas (NADH dehydrogenase subunit 1 [*ND1*]: *P* = 1.79×10^−3^; cytochrome c oxidase subunit 1 [*COX1*]: *P* = 1.54×10^−3^; NADH dehydrogenase subunit 1, [*ND6*]: *P* = 4.62×10^−3^). The latter may be the result of increased expression levels of mtDNA transcription-related genes mitochondrial RNA polymerase (*POLRMT)* (*P* = 2.55×10^−4^) and transcription factor B2 mitochondrial (*TFB2M)* (*P* = 7.88×10^−5^). KC corneas also had increased mtDNA damage (*P* = 3.63×10^−10^), higher ROS levels, and lower mitochondrial membrane potential and ATP levels compared with normal corneas. Decreased integrity, content and increased transcript level of mtDNA are associated with KC. These changes may affect the generation of ROS and play a role in the pathogenesis of KC.

## Introduction

Keratoconus (KC) is a degenerative corneal disease, which is characterized by corneal ectasia, thinning, and cone-shaped protrusion, resulting in reduced vision, irregular astigmatism, and corneal scarring [1, 2]. It is a significant clinical problem worldwide, affecting both genders and all ethnicities [3, 4]. Owing to the limited availability of medical treatments, end-stage KC patients have to accept corneal transplantation.

The etiology of KC is poorly understood. Despite extensive research [4], the molecular pathogenesis of KC remains unknown in the majority of patients. Studies conducted in the early 1990s suggested that KC corneas suffered oxidative damage and that they had abnormal level of stress-related enzymes [5, 6], indicating that oxidative stress (OS) may play a role in the pathogenesis of KC [7, 8]. Oxidative phosphorylation in mitochondria is the major source of endogenous reactive oxygen species (ROS) [9]. Mitochondria have their own genome, mitochondrial DNA (mtDNA), which encodes 13 subunits of respiratory complexes I, III, IV, and V [10, 11]. As mtDNA is closely related with mitochondrial function, the mtDNA content, integrity, and transcript levels may affect the generation of ROS and be involved in the pathogenesis of KC. In a previous study, we showed that there was a significant decrease in the leukocyte mtDNA content of KC patients compared to that of control subjects [12]. In an American population, Atilano et al. reported that KC corneas had a lower mtDNA-to-nDNA (nuclear DNA) ratio and more mtDNA damage than do normal corneas [13]. These results suggest that mtDNA variations may be involved in the pathogenesis of KC, but as of yet no one had attempted to study the relationship between mtDNA and KC systematically in order to uncover the underlying mechanisms. Therefore, to further validate these results in larger cornea samples and study the underlying mechanisms, we carried out this study.

Hundreds to thousands of copies of mtDNA exist in each cell. Accumulating evidence has shown that mtDNA content control is an important aspect of mitochondrial genetics and biogenesis, and is essential for normal cellular function [14, 15]. In eukaryotic cells, mtDNA is replicated by mtDNA polymerase [16, 17]. The polymerase (DNA directed), gamma (*POLG*) gene (nDNA-encoded) encodes catalytic subunit of mtDNA polymerase, and plays a key role in mtDNA replication [18]. The mitochondrial transcription factor A (*TFAM*, nDNA-encoded) gene encodes a key mitochondrial transcription factor, which functions in mtDNA replication and regulates the mitochondrial genome content [19]. To further validate the relationship between mtDNA content and KC, we measured the relative mtDNA content and transcript levels of key genes (*POLG* and *TFAM*) related to mtDNA replication in KC and normal corneas.

Two promotors, heavy strand 2 and light strand, transcribe the entire heavy strand and light strand, respectively [20]. The process of transcription initiation in mitochondria involves three types of proteins: mitochondrial RNA polymerase (POLRMT, nDNA-encoded), TFAM (nDNA-encoded), and mitochondrial transcription factors B1 and B2 (TFB1M, TFB2M; nDNA-encoded) [21]. To investigate the relationship between mtDNA transcription and KC, and uncover the underlying reasons, we measured the transcript levels of genes located in the mtDNA light strand *(*NADH dehydrogenase subunit 6, *ND6;*mtDNA-encoded), heavy strand *(*NADH dehydrogenase subunit 1 (*ND1*, mtDNA-encoded*)* and cytochrome c oxidase subunit 1 (*COX1*, mtDNA-encoded)), as well as those of, key genes (*POLRMT*, *TFAM*, and *TFB2M;* nDNA-encoded) related to mtDNA transcription in the samples [22]. As the integrity of mtDNA is very important for mitochondrial function, and mtDNA damage is a source of OS, the levels of mtDNA damage in KC corneas were also examined.

In short, we designed a case-control study to research the relationship between mtDNA integrity, content, transcription, and KC comprehensively for the first time, in an attempt to study the relationship between mtDNA and KC systematically and uncover the underlying mechanisms.

## Materials and Methods

### Ethics Statement

The study was performed in accordance with the Declaration of Helsinki and approved by the Ethics Committee of Shandong Eye Institute (Qingdao, China). Written informed consent was obtained from all the participants (or guardians).

### Samples

This study included 106 normal corneas (mean age, 38 years; range, 10–76) and 198 KC corneas (mean age, 21 years; range, 13–39). The surplus normal corneas after penetrating keratoplasty were obtained from the Eye Bank of Qingdao Eye Hospital. To ensure the consistency of the corneal regions studied in both groups, we just collected the normal corneas with larger area of surplus. The KC corneas were collected from ophthalmologists of Qingdao Eye Hospital and Shandong Eye Hospital after surgery. The diagnosis of KC was based on clinical examination (corneal stromal thinning, Vogt’s striae, Fleischer’s ring, Munson’s sign, signs of videokeratography, and refractive errors). The KC and control corneas were harvested and handled in a similar manner. Three fresh normal corneas and three fresh KC corneas were used for cell culture immediately after surgery. Two fresh normal corneas and two fresh KC corneas were used for western blot analyses. In the remaining (including 101 normal corneas and 193 KC corneas), some of the corneas (37 normal corneas and 37 KC corneas) were cut into halves and stored under appropriate condition for DNA and RNA extraction respectively. The others corneas were stored at -80°C for DNA extraction immediately after surgery.

### Cell culture and fluorescent staining

Normal human corneal fibroblast (HCF) and keratoconus corneal fibroblast (KCF) cells were isolated from three normal and three KC corneas as described previously [23], and cultured in DMEM/F12 medium (Corning, USA) containing 10% fetal bovine serum (FBS) (Gibco, USA) at 37°C with 5% CO_2_. The cells were collected after 48 h of P1 generation for ROS, mitochondrial membrane potential, ATP measurement and mitochondria staining. The ROS levels were measured using an ROS Assay Kit (Beyotime, China). Mitochondrial membrane potential was measured using a JC-1 Assay Kit (Beyotime, China). ATP levels were measured using an ATP Assay Kit (Beyotime, China) and Mito-Tracker Green (Beyotime, China) was used to label mitochondria. All the fluorescent stainings were processed according to the manufacturer’s protocols, and the results were observed using a laser scanning confocal microscope or an automatic microplate reader. For ROS, mitochondrial membrane potential and Mito-Tracker Green, each group was photographed, and the images were imported into Image J software (National Institutes of Health, Bethesda). The fluorescence intensity of each group was quantified and analyzed using Image J software.

### Western blot

Total protein was prepared from each cornea using radioimmunoprecipitation assay (RIPA) buffer (Galen, Beijing, China) and quantified. Western blot analyses were performed as we described previously [24]. For each sample, the levels of proteins of interest were normalized to that of glyceraldehyde-3-phosphate dehydrogenase (GAPDH). Primary antibodies included dynamin-related protein 1 (DRP1) antibody (ab184247, Abcam), superoxide dismutase 2 (SOD2) antibody (ab68155, Abcam), and GAPDH antibody (KC-5G5, Kangchen, Shanghai, China).

### Measurement of mtDNA content

In total, 101 normal corneas and 193 KC corneas were processed for DNA extraction. Total DNA was isolated from the corneas using the standard phenol/chloroform method. The DNA concentration was measured by BioPhotometer (Eppendorf, Hamburg, Germany). The relative mtDNA content was measured by fluorescence-based quantitative real-time PCR (qPCR) and the 2^-ΔΔCT^ method, as described in previous studies [12, 25]. The primer pair L375 (5’-CACCAGCCTAACCAGATTTC-3’) and H475 (5’-GGGTTGTRTTGATGAGATTAGT-3’) was used for mtDNA detection. The amplification of the single copy nuclear b-globin gene (HBg1: 5’-GCTTCTGACACAACTGTG TTCACTAGC-3’; HBg2: 5’-CACCAACTTCATCCACGTTCACC-3’) was used for normalization. The values were expressed as mtDNA/nDNA ratios.

### mtDNA transcript level and detection of the expression of related genes

In total, 37 normal corneas and 37 KC corneas were processed for RNA extraction and used to measure the transcript levels of mtDNA and related genes. Total RNA was isolated (NucleoSpin RNA II System; Macherey-Nagel, Duren, Germany) and quantified using a spectrophotometer (BioPhotometer; Eppendorf, Hamburg, Germany). cDNA was synthesized from RNA using a commercial kit (PrimeScript ^™^ RT Reagent Kit (Perfect Real Time); Takara, Dalian, China). The expression levels of *POLG* (nDNA-encoded), *TFAM* (nDNA-encoded), *ND6* (mtDNA-encoded), *ND1* (mtDNA-encoded), *COX1* (mtDNA-encoded), *POLRMT* (nDNA-encoded), and *TFB2M* (nDNA-encoded) genes were measured by qPCR and normalized to glyceraldehyde-3-phosphate dehydrogenase (*GAPDH*). The primer sequences of the genes used for qPCR are shown in Table 1.

**Table 1:** Primer sequences of the genes used for qPCR.

Genes	Forward	Reverse
***POLG***	5’-CAACCCCTAGCTCTGACTGC-3’	5’-CCACGTCGTTGTAAGGTCCA-3’
***TFAM***	5’-CGCTCCCCCTTCAGTTTTGT- 3’	5’-CCAACGCTGGGCAATTCTTC-3’
***ND6***	5’-TCAACGCCCATAATCATACAAA-3’	5’-GATGGCTATTGAGGAGTATCCTGAG-3’
***ND1***	5’-ACTACGCAAAGGTCCCAACG-3’	5’-GGCGGGTTTTAGGGGTTCTT-3’
***COX1***	5’-TTAGCTGACTCGCCACACTC-3’	5’-GGCCACCTACGGTGAAAAGA-3’
***POLRMT***	5’-GGGACCATCGAAAGGTGTCT-3’	5’-CTTCAGAACAGTGGCCCGAT-3’
***TFB2M***	5’-TCCCGGAAATCCAGACTTGT-3’	5’-TGATGACCAAGGCTCCATGTG-3’
***GAPDH***	5’-ACCACAGTCCATGCCATCAC-3’	5’-TCCACCACCCTGTTGCTGTA-3’

### Long Extension PCR (LX-PCR)

DNA samples from 101 normal corneas and 193 KC corneas were used to detect mtDNA damage. LX-PCR was performed as described previously with a few modifications [26]. The primer pair LXF (5’-TGAGGCCAAATATCATTCTGAGGGGC-3’) and LXR (5’-TTTCATCATGCGGAGATGTTGGATGG-3’) was used to amplify the mtDNA genome. Twenty microliter reactions contained 1× GC buffer I (Mg^2+^ Plus), 10 ng DNA, 0.4 μM each of the primers, 0.4 mM each of deoxynucleotide triphosphate, and 1 U of TaKaRa LA Taq DNA polymerase (Takara, Dalian, China). The reaction mixture was denatured at 95°C for three minutes followed by 35 cycles of 10 seconds at 98°C, 16 minutes at 68°C, and a final elongation for 10 minutes at 72°C. The LX-PCR products were separated by electrophoresis on a 0.8% agarose gel stained with ethidium bromide.

### Statistical analysis

Previous studies reported an inverse association between the mtDNA content and age [27, 28], with mitochondrial dysfunction, mtDNA damage, and ROS increasing with age [29, 30]. An analysis of covariance was used to determine the differences in the relative mtDNA content and mtDNA damage of the KC and normal corneas, adjusting for the effect of age. A nonparametric test was used to determine the differences in transcript levels of mtDNA and related genes and the fluorescence intensity between the KC and normal corneas. Spearman’s rank correlation analysis was used to evaluate the relationships between different parameters. All the statistical analyses were performed using SPSS 17.0 (SPSS Inc., USA). Statistical significance was defined as α = 0.05.

## Results

### KCF cells had increased ROS production, weaker mitochondrial fluorescence intensity, and decreased mitochondrial membrane potential, ATP levels

**Fig 1:**
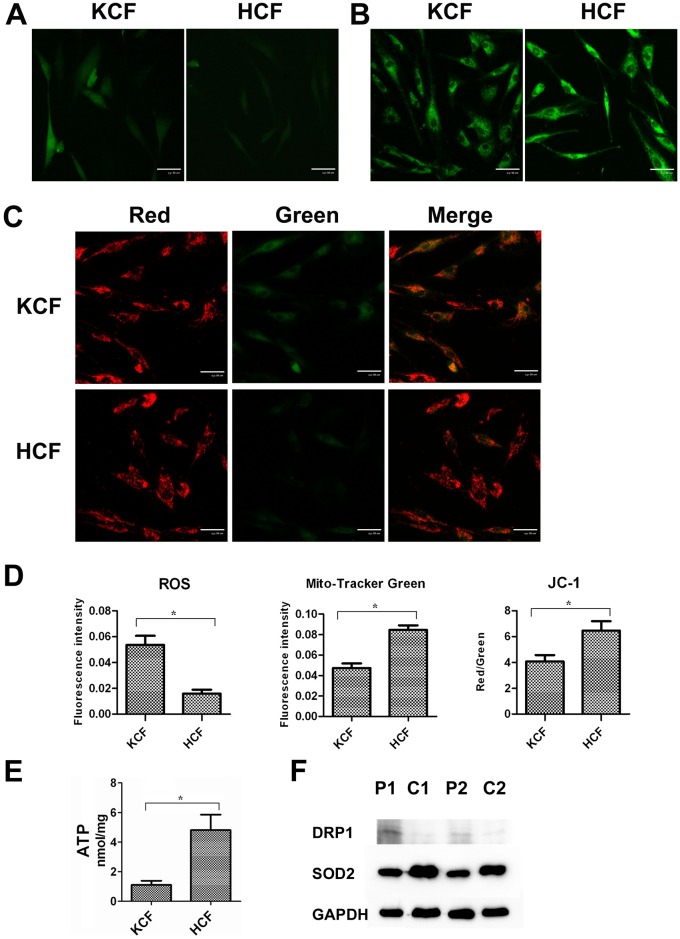
The inmunofluorescence staining results of HCF and KCF cells. A, reactive oxygen species (ROS); B, mitochondria labeled by Mito-Tracker Green; C, mitochondrial membrane potential measured by the JC-1 Assay Kit. Cells with high mitochondrial membrane potential showed enhanced red fluorescence. In contrast, cells with low mitochondrial membrane potential showed only green fluorescence. Differences in the ratio of red to green fluorescence are representative of changes in mitochondrial membrane depolarization. D, The fluorescence intensity of each group (n = 3) quantified by Image J software. E, ATP levels (n = 3); F, western blot analysis of DRP1 and SOD2. P1and P2: KC corneas; C1and C2: normal corneas.

The fluorescence staining results of the normal and KCF cells are shown in Fig 1A–1E. The KCF cells had significantly higher ROS levels compared to the normal corneal fibroblast cells (Fig 1A and 1D). The fluorescence intensity of the Mito-Tracker-labeled mitochondria in the KCF cells was significantly weaker than that of the normal corneal fibroblast cells (Fig 1B and 1D). Similarly, the mitochondrial membrane potential and ATP levels of the KCF cells were significantly lower than that of the normal corneal fibroblast cells (Fig 1C–1E). These results showed that KCF cells had greater ROS production, lower mitochondrial fluorescence intensity, and lower mitochondrial membrane potential and ATP levels compared to normal corneal fibroblast cells. In addition, the western blot results showed that KC corneas have higher DRP1 expression and lower SOD2 expression compared to normal corneas (Fig 1F).

### Decreased mtDNA content and expression of genes involved in replication in KC corneas

**Fig 2:**
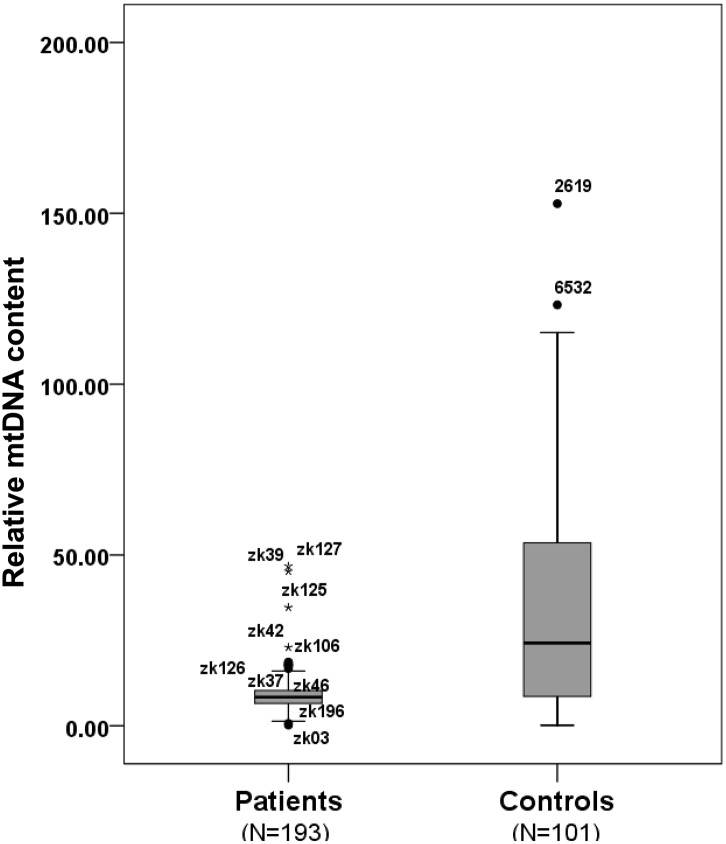
Relative mtDNA content in 101 normal corneas and 193 KC corneas. The medians and the 25th and 75th percentiles of relative mtDNA content in the KC and normal corneas are shown.

To test whether variations in the mtDNA content were associated with KC, we measured the mtDNA content in 193 KC corneas and 101 normal corneas. The results are shown in Table 2. There was a significant decrease of mtDNA content in KC corneas compared to normal corneas (*P* = 9.19×10^−24^). The medians and the 25th and 75th percentiles of relative mtDNA content of KC and normal corneas are shown in Fig 2.

**Table 2:** Mitochondrial DNA content and expression of genes involved in mtDNA replication and transcription in the corneal samples of the patients and controls.

Parameters/Genes	Patients ^a^	Controls ^a^	*P* Value
**mtDNA content**			
**Total**	9.315±0.389	36.904±3.533	9.19×10^−24^
**Subgroup**	5.238±0.238	72.605±4.955	1.62×10^−17^
**Transcript level of genes involved in mtDNA replication**			
***POLG***	1.023±0.136	0.950±0.121	8.51×10^−1^
***TFAM***	2.928±0.778	6.792±1.659	3.26×10^−3^
**Transcript level of L strand**			
***ND6***	0.744±0.275	0.209±0.038	4.62×10^−3^
**Transcript level of H strand**			
***ND1***	1.120±0.273	0.445±0.072	1.79×10^−3^
***COX1***	1.179±0.210	0.609±0.094	1.54×10^−3^
**Transcript level of genes involved in mtDNA transcription**			
***POLRMT***	1.173±0.102	0.641±0.077	2.55×10^−4^
***TFB2M***	1.178±0.121	0.526±0.074	7.88×10^−5^

Note. The values are given as means ±standard errors.

^a^, including the 37 KC corneas and 37 normal corneas except the “total” group, which included 193 keratoconus corneas and 101 normal corneas.

**Fig 3:**
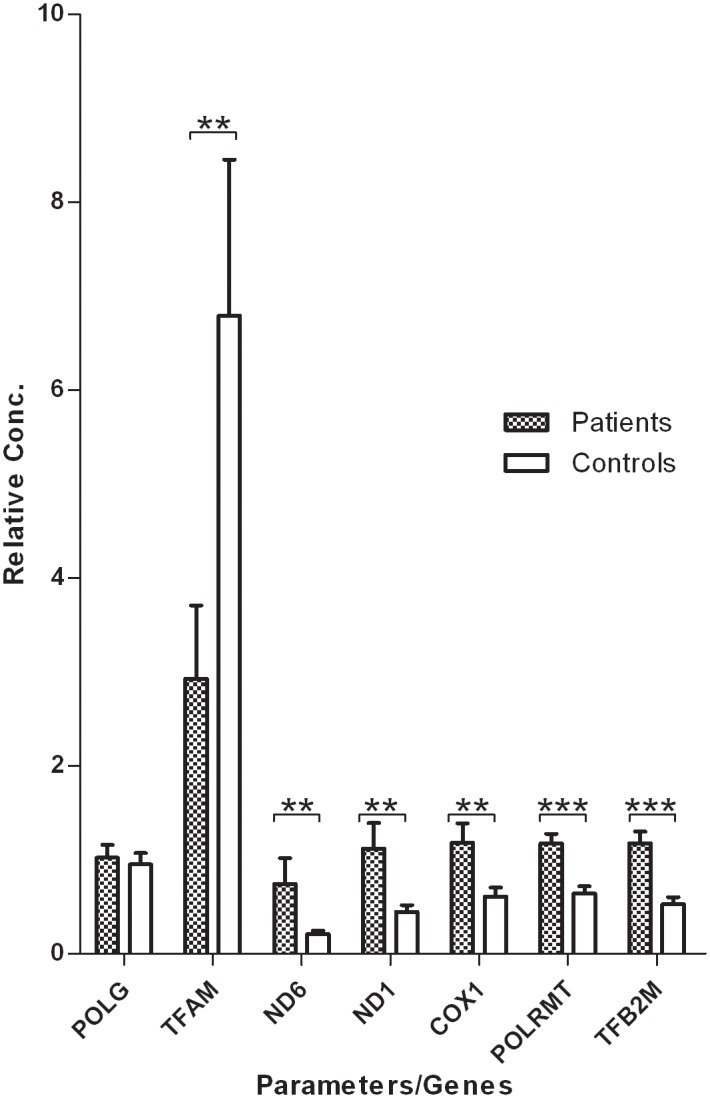
The expression of genes involved in mtDNA replication and transcription in each of the 37 corneal samples of the patients and controls. **, *P*<0.01; ***, *P*<0.001.

To investigate the relationship between the replication-related genes *POLG* and *TFAM* and relative mtDNA content, the transcript levels of these genes were measured in 37 KC and 37 normal corneas. The results revealed no significant difference in the transcript level of the *POLG* gene between the KC corneas and normal corneas (Table 2, Fig 3). However, the *TFAM* transcript levels of the KC corneas were significantly lower than those of the normal corneas (*P* = 3.26×10^−3^) (Table 2, Fig 3). The *TFAM* transcript levels also showed a significant positive correlation with the mtDNA content (Correlation coefficient = 0.372, *P* = 2.31×10^−3^) (Table 3).

**Table 3:** Spearman’s correlation coefficients between the mtDNA content, damage and genes involved in mtDNA replication and transcription in the corneal samples of the patients and controls.

Correlation coefficient	mtDNA content	*POLG*	*TFAM*	*ND6*	*ND1*	*COX1*	*POLRMT*	*TFB2M*	mtDNA damage
**mtDNA content**	1.000	0.018	0.372**	-0.346**	-0.332**	-0.363**	-0.349**	-0.519***	-0.289***
***ND6***	-0.346**	0.077	-0.274*	1.000	0.815***	0.797***	0.672***	0.730***	0.366**
***ND1***	-0.332**	0.109	-0.156	0.815***	1.000	0.946***	0.716***	0.724***	0.443***
***COX1***	-0.363**	0.155	-0.147	0.797***	0.946***	1.000	0.723***	0.746***	0.390***

Note.

*, *P*<0.05;

**, *P*<0.01;

***, *P*<0.001.

### Increased transcript levels of mtDNA and transcription related genes in KC corneas

The transcript levels of *ND6* (locus in mtDNA light strand, mtDNA-encoded), *ND1* and *COX1* (locus in mtDNA heavy strand, mtDNA-encoded) were measured in 37 KC and 37 normal corneas. The mtDNA transcript levels of the transcription related genes *POLRMT* (nDNA-encoded) and *TFB2M* (nDNA-encoded) were also detected in the same samples. The results are shown in Table 2 and Fig 3. The *ND6* expression levels was significantly higher in the KC corneas than the normal corneas (Table 2, Fig 3). Similarly, the *ND1* and *COX1* expression levels of the KC corneas were significantly higher than those of normal corneas (Table 2, Fig 3). The transcript levels of *ND6*, *ND1* and *COX1* were all significantly negatively correlated with the mtDNA content (Table 3). The expression of the transcription related genes *POLRMT* and *TFB2M* was significantly increased in KC corneas compared to normal corneas (Table 2, Fig 3), and the expression was significantly positively correlated with the transcript levels of mtDNA (Table 3, all *P* values <1×10^−3^).

### Increased mtDNA Damage in KC Corneas

**Fig 4:**
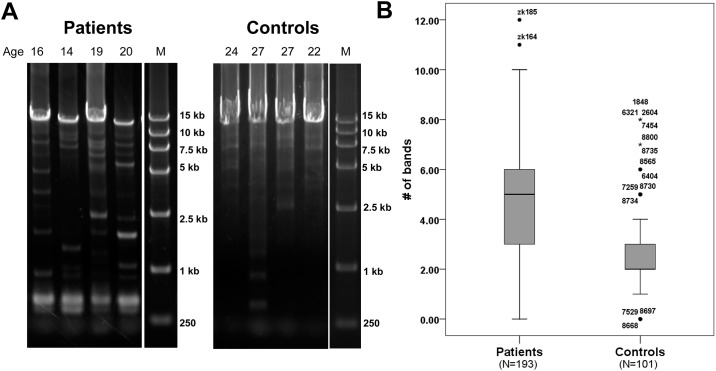
Increased mtDNA damage in KC corneas. A, Representative agarose gels image of mtDNA damage in the KC patients and controls. B, Quantitation of smaller-sized bands per individual in normal and KC corneal LX-PCR mtDNA. The medians and the 25th and 75th percentiles of smaller-sized mtDNA bands of the KC and normal corneas are shown.

LX-PCR was used to detect mtDNA damage, as previous described. The major LX-PCR product representing full-length mtDNA is 16.2 kb. When nucleotide substitutions, rearrangements, or deletions are present in the mtDNA template, LX-PCR yields smaller-sized products. We did LX-PCR analysis in 193 KC corneas and 101 normal corneas. Smaller-sized mtDNA bands (<16.2 kb and >1 kb) in the KC and normal samples were quantified. The mean number of smaller-sized mtDNA bands in the KC and normal corneas was 4.660 (standard error (SE) = 0.176) and 2.634 (SE = 0.182), respectively. There was a significant increase in the number of smaller-sized mtDNA bands in the KC corneas compared to the normal corneas (*P* = 3.63×10^−10^). The medians and the 25th and 75th percentiles of the smaller-sized mtDNA bands of KC and normal corneas are shown in Fig 4. The smaller-sized mtDNA bands also showed a significant negative correlation with the mtDNA content (correlation coefficient = -0.289, *P* = 5.70×10^−7^). For the subgroup (i.e., the 37 KC and 37 normal corneas), the mean number of smaller-sized mtDNA bands in the KC and normal corneas was 4.972 (SE = 0.426) and 2.417 (SE = 0.212), respectively. There was a significant increase in the number of smaller-sized mtDNA bands in the KC corneas compared to the normal corneas in the subgroups (*P* = 4.82×10^−5^). According to the subgroup data, the smaller-sized mtDNA bands showed a significant positive correlation with mtDNA transcription (Table 3).These results showed that the KC corneas had significantly decreased mtDNA content and increased mtDNA transcription and damage relative to normal corneas.

## Discussion

Previous studies found that KCF cells had increased ROS and lower mitochondrial membrane potential compared with normal corneas [7, 8, 31], which indicated that these components may play a part in the pathogenesis of KC [32]. To validate these results, we repeated the experiments performed in those studies using KC and normal corneas and obtained consistent findings. In the present study, KCF cells had more ROS products, and lower mitochondrial membrane potential and ATP levels compared with normal corneal fibroblasts, suggesting that mitochondrial function plays a role in the pathogenesis of KC. In addition, we also found that KC corneas have higher DRP1 expression and lower SOD2 expression than normal corneas. DRP1 is a fundamental component of mitochondrial fission, and fission results in fragmented mitochondria more capable of producing of ROS [33]. SOD2 is a member of the iron/manganese superoxide dismutase family, and plays an antiapoptotic role against oxidative stress [34]. Higher DRP1 expression and lower SOD2 expression in KC corneas verified again that the oxidative stress is involved in KC, and plays an important role in the pathogenesis of KC.

Accumulating evidence has shown that mtDNA content control is very important for mitochondrial biogenesis and normal cellular function [14, 15]. Previous studies observed that the mtDNA content is associated with many eye diseases other than KC, including autosomal dominant optic atrophy, diabetic retinopathy, and Leber's hereditary optic neuropathy [35–37]. Atilano et al. found that KC corneas (N = 16) had lower mtDNA-to-nDNA ratios than normal corneas (N = 10) [13]. However, their results showed this trend with an insignificant *P* value (*P* < 0.7). In the present study, we measured the relative mtDNA content in a larger sample size and found a significant decrease of mtDNA content in KC corneas compared to normal corneas. This result suggested that decreased mtDNA content is associated with KC. In the present study, there was no significant difference in *POLG* expression levels between the KC and normal corneas, but that *TFAM* expression levels of the KC corneas were significantly lower than those of the normal corneas. The *TFAM* expression levels also showed a significant positive correlation with the mtDNA content. The findings suggest that the lower relative mtDNA content of KC corneas may be due to decreased *TFAM* gene expression.

The transcript level of mtDNA is crucial for the function of the oxidative phosphorylation. Chwa et al. reported that the mitochondrial cytochrome oxidase subunit 2 RNA levels were increased in the KC fibroblasts cultured at pH 7 compared with the normal cultures, and mitochondrial cytochrome oxidase subunit 1 RNA levels were elevated in KC cells but without reaching significance [31]. In the current study, we further found that the transcript levels of mtDNA were significantly increased in KC corneas compared with normal corneas, and the expressions of the transcription-related genes *POLRMT* and *TFB2M* were significantly increased in KC corneas and positively correlated with the transcript levels of mtDNA. These results suggested that an increased transcript level of mtDNA is associated with KC, which may be due to increased *POLRMT* and *TFB2M* gene expression.

The integrity of mtDNA is very important for mitochondrial function, and mtDNA damage is a source of OS. The human cornea is exposed to oxidizing UV and blue light, and contains the highest amount of mtDNA fragment deletions compared to other tissues of the eye [38, 39]. Increased mtDNA damage is reported to be involved in many OS related eye diseases, including age-related macular degeneration, diabetic retinopathy, age-related cataracts, and KC [13, 32, 40–42]. OS can cause DNA damage [43]. As a major producer of ROS, mitochondria may thus be subjected to direct attack by ROS [44]. mtDNA is particularly prone to oxidative damage compared to nuclear DNA; because of its proximity to the ROS-generating respiratory chain, it is directly exposed to ROS and inefficient DNA repair systems [45]. Conversely, mtDNA damage is also a source of OS. mtDNA damage could lead to progressive respiratory chain dysfunction and a further increase in ROS production as a consequence of this dysfunction [43]. Atilano et al. reported that KC corneas (N = 16) exhibit more mtDNA damage than do normal corneas (N = 10) in the American KC population [13]. To further validate this result in Chinese population, we detected the mtDNA damage in a larger sample size, and found that there was a significant increase of mtDNA damage in KC corneas compared to normal corneas. This result suggested that increased mtDNA damage is associated with KC.

In summary, the following results were obtained in this study. The mtDNA content of KC corneas was lower than that of normal corneas, possibly due to decreased *TFAM* gene expression. However, the transcript levels of mtDNA were significantly increased in KC patients corneas compared to normal corneas. In addition, the expression levels of the transcription related genes *POLRMT* and *TFB2M* were significantly increased in KC patients’ corneas, and they were positively correlated with the transcript levels of mtDNA. KC corneas also showed more mtDNA damage, higher ROS levels, and lower mitochondrial membrane potential and ATP levels compared to normal corneas. mtDNA damage showed a significant negative correlation with mtDNA content. In addition, mtDNA transcript levels were significantly negatively correlated with mtDNA content and positively correlated with mtDNA damage. Based on the results of the present study, we speculate that the mechanism underlying the role of mtDNA in KC may proceed as follows: KC corneas have higher transcript level of mtDNA, possibly due to increased expression of the *POLRMT* and *TFB2M* genes. A combination of higher transcript level of mtDNA and higher mtDNA damage could lead to progressive respiratory chain dysfunction and a further increase in ROS production as a consequence of this dysfunction [32]. Decreased mtDNA content caused by reduced *TFAM* gene expression may lead to increased proportion of mtDNA damage and reduced quantities of normal mtDNA in the corneal stroma, further increasing ROS formation and additional OS in cornea. However, this speculation needs further validation and a detailed assay of cornea samples, such as Northern blots of mtDNA-transcribed mRNAs or other functional analysis.

## Conclusions

In conclusion, we found that decreased integrity, content and increased transcript level of mtDNA is associated with KC, and these changes may affect the generation of ROS and be involved in the pathogenesis of KC.

## Supporting Information

S1 Table Relative mtDNA content in the 193 KC corneas and 101 normal corneas. (XLS)

S2 Table Expression of genes involved in mtDNA replication and transcription in corneal samples of the patients and controls. (XLS)

S3 Table Smaller-sized mtDNA bands in 193 KC corneas and 101 normal corneas. (XLS)
